# Ants Response to Human-Induced Disturbance in a Rain Tropical Forest

**DOI:** 10.1007/s13744-018-0624-5

**Published:** 2018-08-23

**Authors:** B Walter, A Graclik, P Tryjanowski, O Wasielewski

**Affiliations:** Institute of Zoology, Poznan Univ of Life Sciences, Wojska Polskiego 71 C, 60-625 Poznan, Poland

**Keywords:** Ants, biodiversity loss, colony size, deforestation, *Platythyrea punctata* tropical rain forest

## Abstract

A high rate of human-induced disturbance of tropical ecosystems results in enormous loss of biodiversity due to local extinctions. Yet, mechanisms at the population level that lead to the extinction are still poorly understood. Here we tested the hypothesis that human-induced disturbance results in smaller amount of nesting sites for wood-dwelling arthropods that leads to smaller population size and diminished reproduction, and therefore, may promote local extinctions. We completed censuses in less-disturbed and human-disturbed secondary rain forest plots in Puerto Rico. We measured population size and brood production in wood-nesting ants and examined whether these parameters differ between less-disturbed and more-disturbed habitats. In addition, we measured volume of wood parts of all inhabited and potential nesting sites to assess nest site availability. We found that more human-disturbed forests furnish smaller nest sites, resulting in diminished population size and lowered brood production. Our study shows that human-induced disturbance decreases volume of available nesting sites that leads to decreased population size and lowered reproduction. Thus, in addition to the well-documented loss of species richness in human-disturbed tropical habitats, we demonstrated the direct effect of the disturbance that may promote vulnerability of local populations.

**Electronic supplementary material:**

The online version of this article (10.1007/s13744-018-0624-5) contains supplementary material, which is available to authorized users.

## Introduction

**Fig 1:**
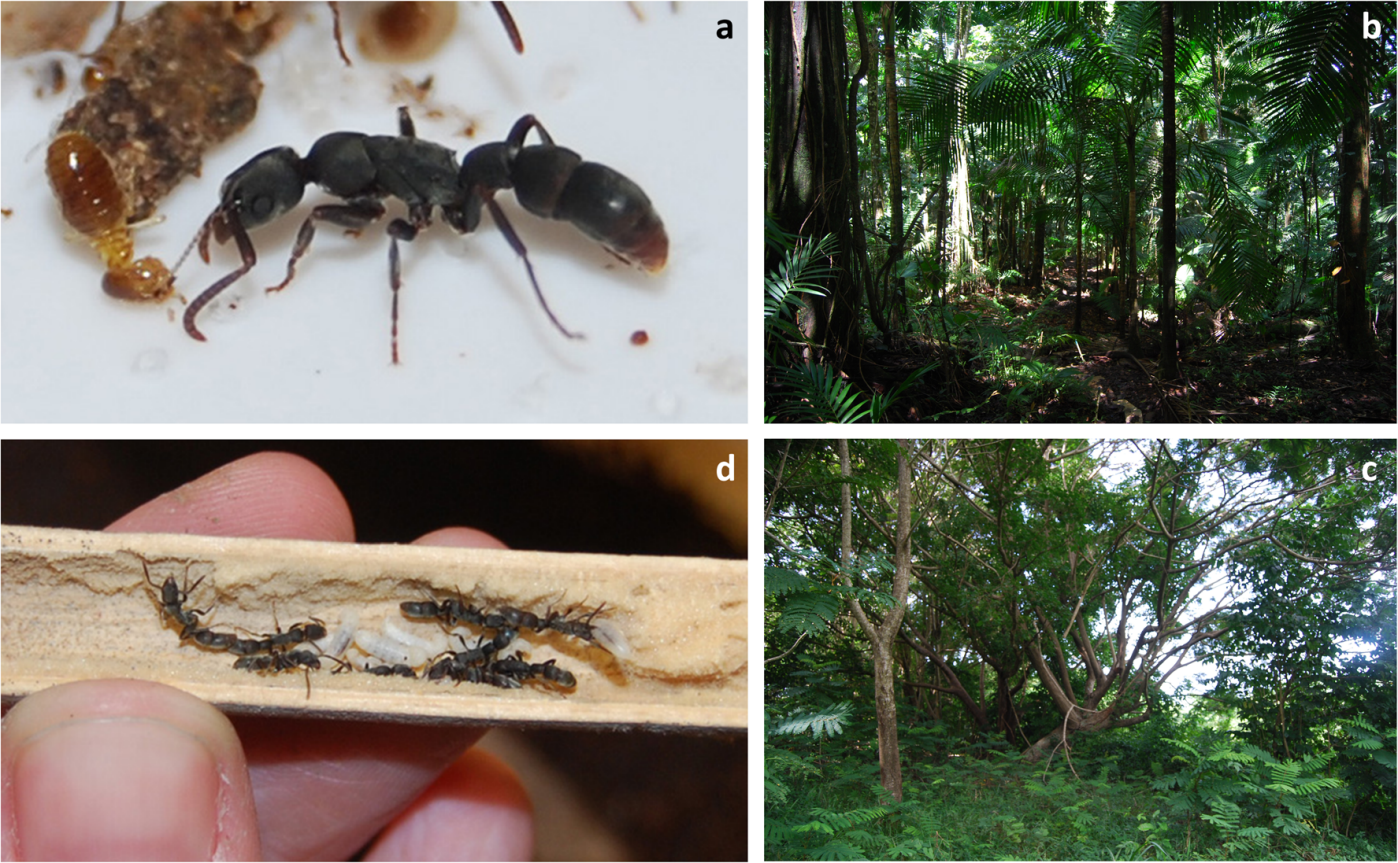
Tropical rain forests in the Caribbean: less disturbed (**B**) and more disturbed (**C**) by human activity. More-disturbed forests furnish smaller wood pieces, such as logs or branches, which results in smaller size of nesting sites (e.g., artificial laboratory nest similar to the natural) (**D**) and directly leads to diminished population size and lower brood production in wood-nesting *Platythyrea punctata* ants (**A**).

Tropical rain forests are the most complex ecosystems and host the highest number of species on Earth (Myers *et al* 2000, Brooks *et al* 2006, Gibson *et al* 2011). Unfortunately, the last four decades were marked by an enormous scale of deforestation caused by human activity (Skole & Tucker 1993, Achard *et al* 2002, Primack & Corlet 2005). Although vegetation often forms secondary rain forest on previously deforested areas (Achard *et al* 2002, Chazdon 2008), such human-disturbed habitats are characterized by substantially diminished biodiversity, as compared with primary forest (Bradshaw *et al* 2008, Chazdon 2008, Gardner *et al* 2008). The impoverished biodiversity of disturbed habitats is linked directly to simplified structural complexity and loss of resources, and as a consequence, indirectly to changes in microclimate and habitat availability (Tews *et al* 2004, Klimes *et al* 2012). Although the effect of human-induced disturbance on species richness has already been documented in various plant and animal taxa (Roth *et al* 1994, Kalif *et al* 2001, Dunn 2004), little is known about how the human disturbance influences reproduction and population size of species, which may lead to local extinction, resulting in the lower biodiversity of disturbed tropical forests (Furrer & Pasinelli 2016, Alroy 2017). One of the major indicators of tropical biodiversity are ants, constituting up to 85% of animal biomass in tropical rain forest canopy (Holldobler & Wilson 1990, Floren & Linsenmair 1997, Davidson *et al* 2003). Following the general trends, ant species richness is substantially impoverished in habitats disturbed by human activity (Kalif *et al* 2001, Klimes *et al* 2012). Although the lower ant species number may result from a reduced availability of nest sites in human-disturbed secondary forests (Shulz & Wagner 2002, Armbrecht *et al* 2004, Philpott & Foster 2005, Powell *et al* 2011, Klimes *et al* 2012*,* Yanoviak *et al* 2012), the direct response of insect populations to human-induced disturbance that promotes local extinction is still poorly understood (Zmichorski 2011, Santos 2016). Forest disturbance may reduce nest site availability because during forest cutting in tropics, litter and organic matter are immediately destroyed (Lohbeck *et al* 2015, da Silva *et al* 2017). The disturbance may lead to diminished size of population and individuals or colonies that results in impoverished brood production. Similar effect of the human disturbance was found in neotropical dung beetles for which human activity leads to decrease population size and lower individual body mass (Gardner *et al* 2008). In addition, we expected for social insects that the disturbance will result in a smaller colony size. In social insects, brood productivity is strictly linked with colony size: larger colonies produce larger brood number and larger proportion of sexual brood that later may establish new colonies (e.g., Walter *et al* 2011, Luque *et al* 2013, DiRienzo & Dornhaus 2017). Therefore, the predicted smaller colony size may lead to lower brood production resulting in lower viability of populations in more-disturbed locations. In this study, we examined direct mechanisms that affect colony architecture (size, composition, and productivity) of the neotropical ponerine ant *Platythyrea punctata* (Smith 1858) (Fig 1a) by comparing populations from habitats with different degrees of disturbance: less-disturbed mature rain forest versus populations from recently more-human-disturbed rain forest in Puerto Rico (Fig 1b, c). This ant species provides an ideal model to study the effect of habitat change on population parameters, as *P. punctata* occurs both in natural and in disturbed habitats distributed over West Indies and Central American mainland (Seal *et al* 2011). Similar to most of tropical ant species and many other arthropods, the ant *P. punctata* inhabits nest sites inside dead, dry, and soft wood such as twigs, branches, or trunks previously hollowed by bark beetles (Fig 1d; Schilder *et al* 1999, Seal *et al* 2011). Thus, *P. punctata*, like many other tropical arthropods nesting almost exclusively inside wood, is sensitive to human-induced disturbance that removes wood from a habitat, such as logging or clear-cutting. Therefore, we tested hypotheses that (1) colonies from more-disturbed habitats are characterized by smaller size and lower brood production than colonies from undisturbed habitats and (2) differences in colony size and brood production between colonies from less-disturbed and more-disturbed habitats are associated with differences in nest site availability. We indicated the direct mechanism that weakens population structure such as size and reproduction and promotes local extinctions and, as a consequence, biodiversity loss in disturbed locations.

## Material and Methods

### Less-disturbed and more-disturbed habitats

Colonies of the neotropical ponerine ant *P. punctata* were collected in Puerto Rico, West Indies, in the middle of the rain season in August 2011 (Table 1). After collecting, ants were transported to laboratories for detailed studies on their behavior. The distance between study sites was more than 5 km. Firstly, we chose eight plots, 0.25 ha each, in secondary Caribbean hurricane rain forest at El Yunque National Forest that were formerly used as a coffee plantation and have been for natural succession and protected since 1934. The plots at El Yunque National Forest served us as an example of locations without recent human-induced disturbance. As examples of more-disturbed habitats, we chose nine locations across the island, covered by secondary tropical rain forests (Helmer *et al* 2002), where larger trees are removed by logging or are absent due to recent land use for agriculture. The data about recent land-use history were collected from aerial and satellite photos. Vegetation status and type of current human-induced disturbance (i.e., clear-cut or selective logging) were checked during site visits in years 2005 and 2011. Previous studies documented that the populations from examined locations exhibit the same reproduction mode (Kellner & Heinze 2011, Kellner *et al* 2013), i.e., in both types of habitat in Puerto Rico, the *P. punctata* ants reproduce via thelytokous parthenogenesis. Therefore, the differences between populations from less-disturbed and more-disturbed habitats in colony size, brood reproduction, and genetic structure should result from other factors than mode of reproduction.

**Table 1:** Location and differences in number of colonies and individuals between two habitats: less and more disturbed.

Location	Type of habitat	Examined area [ha]	Number of colonies	Number of colony per 0.25 ha	Number of individuals per colony
El Verde*	Less disturbed	3.86	10	0.65	79
El Tunel	More disturbed	0.24	3	3.16	35
Juncos	More disturbed	0.28	6	5.29	36
Liquillo	More disturbed	0.90	18	5.00	29
Parcelas Vieques	More disturbed	1.80	10	1.39	38
Pico	More disturbed	1.57	11	1.75	24
Pitahaya	More disturbed	0.08	1	3.33	46
Sabana	More disturbed	0.27	5	4.61	26
Yuquiyu	More disturbed	0.39	4	2.58	12
Yuquiyu II	More disturbed	0.37	2	1.34	19

*But on this area for more detailed studies were chosen 8 plots each 0.25 by size.

### Colony identification, size, and composition

To identify all colonies from a particular plot, we examined available nest site, such as dead hollowed wood (twigs, branches, logs, trunks) and exceptionally rolled leaves. Although such a direct searching procedure (Bestelmeyer & Casanova 2010) is labor-intensive, it allows to detect nearly all colonies in the study area, regardless of vegetation density. In addition, foragers coming back to the nest with pieces of food were followed. Except for two colonies that were spotted during migration, all colonies in study locations inhabited chambers inside dead and dry hollowed wood. In order to identify the exact colony size (worker number) and number of brood, we gently placed every piece of wood with a nest of *P. punctata* in a plastic box (100 cm long, 50 cm wide, and 25 cm high) with wall edges treated with paraffin oil that prevented ants from escaping. Then we carefully split wood inside the box into ~ 0.5-cm^3^ cubes with a pocket knife, removed wood piece by piece, and transferred all ants and their brood from the box to Falcon vials using an aspirator. After collecting ants from a nest, we waited two additional hours for individuals that were foraging at the time when we removed the nest. Such foragers circulating around the original position of a nest and carrying dead insects were collected. All foragers came back to the nest within one hour, suggesting that our two-hour procedure was sufficient. When all individuals from a single nest were collected, we assessed colony size (number of fully colored workers with black cuticle) and number of brood (larvae, pupae, and freshly eclosed callow workers, distinguished by their pale cuticle).

### Size of inhabited and uninhabited wood

Before wood with a nest site was split to collect a colony, we measured wood length (*l*) and diameter (*d*). Wood size with nest site was calculated as a cylinder with volume defined as *V* = *π* (2 − 1*d*)2. In addition, we measured the volume of uninhabited wood in both less-disturbed and more-disturbed habitats in order to test whether the size of available wood with potential nest sites differs between the habitats. To test these differences, we collected all wood pieces of dry hard wood with cavities bored by other insects. Therefore, the wood that was not inhabited by the ant *P. punctata* was collected from the same plots (described above) that served for collecting ants in less-disturbed and more-disturbed habitats. And for the further analyses, we chose only dry and hard wood with cavities bored by other insects.

### Statistical analyses

All studied variables deviated significantly from normal distribution (Kolmogorov–Smirnov test, *K-S* > 1.41 and *P* < 0.04 in all analyses); therefore, data used in Multiple General Model were *log* transformed in SPSS 21.0 (according to the function *log*_10_ + 1, because of 0 in a few cases) and after transformation followed normal distribution (Kolmogorov–Smirnov test, *K-S* < 1.24 and *P* > 0.09 in all analyses). In other cases, to compare two studied main habitats, the more conservative nonparametric Mann–Whitney *U* test was used. Therefore, the data in comparative analyses were presented as real values, i.e., were not *log*_10_ transformed.

## Results

**Fig 2:**
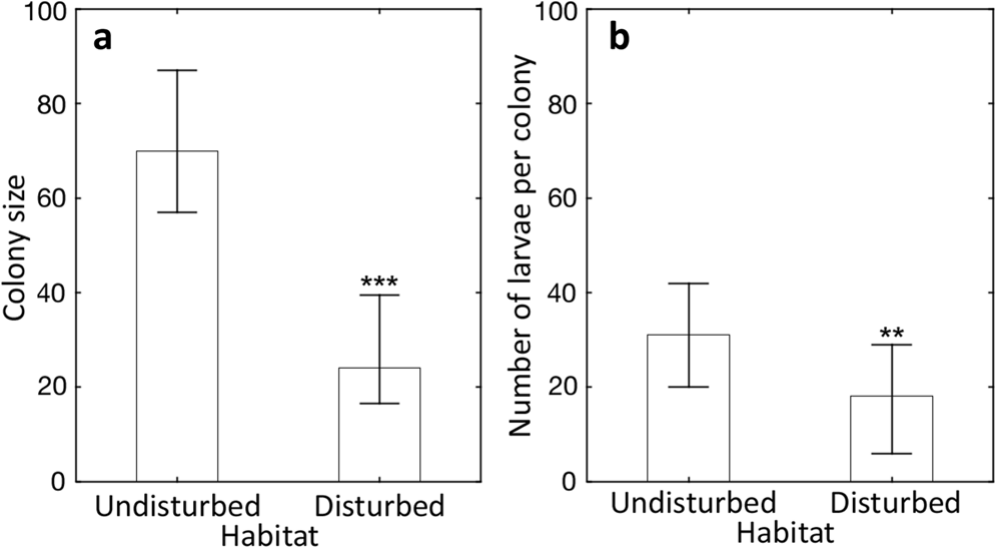
**A**, **B** Colony size (worker number) and number of larvae per colony of the neotropical ponerine ant *Platythyrea punctata* from undisturbed (less disturbed) and human-disturbed (more disturbed) rain forests in Puerto Rico. Columns represent median values; whiskers indicate upper and lower quartiles. Mann–Whitney *U* test: ***P < 0.0001; **P = 0.013 (less disturbed *n* = 17; more disturbed *n* = 30).

**Fig 3:**
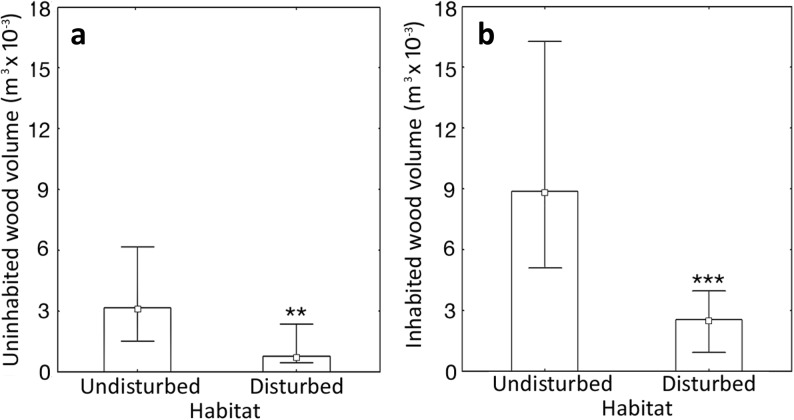
Size of favorable ant nest sites (i.e., dry and hard wood pieces) in undisturbed (less disturbed) and human-disturbed (more disturbed) rain forests in Puerto Rico: **A** volume of wood with cavities not occupied by the Neotropical ponerine ant *Platythyrea punctata*. **B** Volume of wood occupied by the ants. Columns represent median values; whiskers indicate upper and lower quartiles; ***P < 0.001; **P < 0.004 (less disturbed *n* = 17; more disturbed *n* = 30).

**Fig 4:**
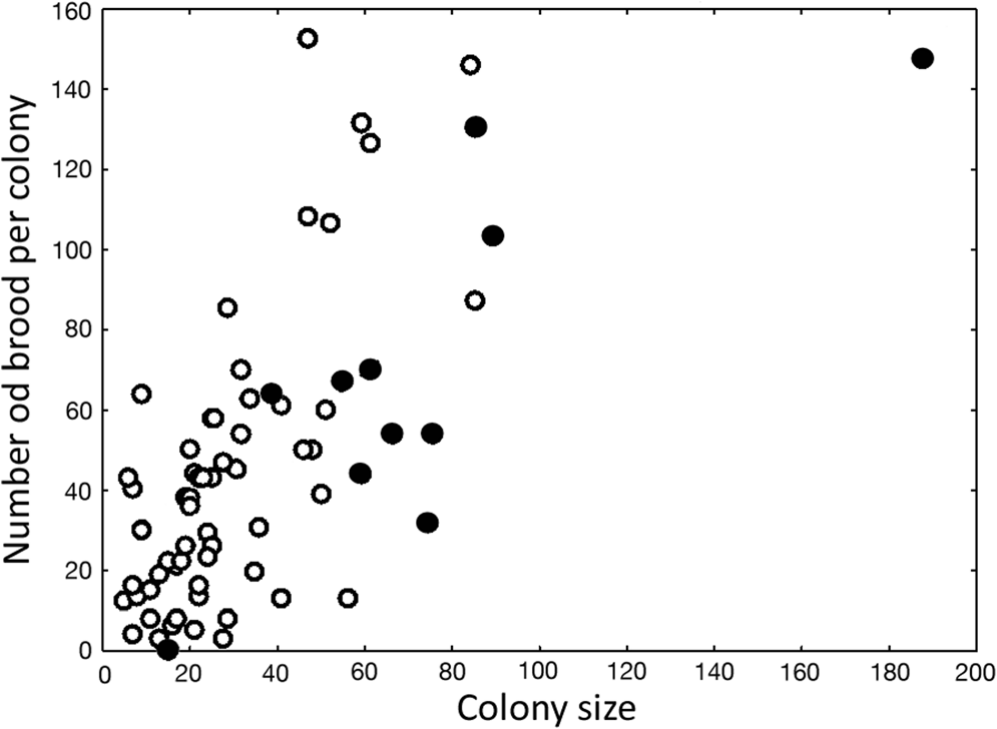
Colony size (worker number) and brood production (sum of larvae, pupae, and freshly eclosed callow workers) in the Neotropical ponerine ant *Platythyrea punctata* from less-disturbed (black circle) and more-human-disturbed (white circle) rain forests in Puerto Rico. Spearman rank order correlation: *n* = 70, *r*_*s*_ = 0.67, *t*(*n* − 2) = 7.36, *P* < 0.00001.

Colonies collected in more-disturbed habitats were on average threefold smaller (Fig 2a, Mann–Whitney *U* test: *U* = 90.0, *Z* = − 4.08; *P* < 0.0001) and had twofold less larvae than colonies from undisturbed habitat (Fig 2b, Mann–Whitney *U* test: *U* = 195.5, *Z* = − 2.49, *P* = 0.013). Larger colony size in the population inhabiting undisturbed habitat can be most likely explained by substantially larger size of nest sites that are available in undisturbed locations. Uninhabited wood with potential nest sites was on average fourfold larger in undisturbed forests than in disturbed forests (Fig 3a, Mann–Whitney *U* test: *U* = 121.0, *Z* = 2.97, *P* < 0.004). Consequently, colonies from less-disturbed forests inhabited on average significantly larger wood pieces than colonies from more-disturbed habitats (Fig 3b, Mann–Whitney *U* test: *U* = 81.5, *Z* = 3.51, *P* < 0.001). As predicted, there was a positive significant correlation between nest site size and colony size (*r* = 0.322, *P* = 0.014) and in the model taking into account both the effect of habitat type (less disturbed vs. more disturbed) and the effect of nest site size; only the effect of habitat was significant (regression analyses: habitat effect *B* = − 43.3 ± 10.1, *P* < 0.0001; nest size *log* volume *B* = 8.3 ± 10.7, *P* = 0.44; interaction between factors *P* = 0.78). We observed that in both less-disturbed and more-disturbed forests, the ant *P. punctata* prefers to nest in hard and dry wood (81% of colonies) rather than soft and wet wood (19% of colonies). These habitat preferences (wood with appropriate size and favorable dryness and hardness (Parr 2012) make the available nest sites for this ant species scarcer. Furthermore, colonies from less-disturbed forests produced significantly more brood than colonies from more-disturbed forests (Mann–Whitney *U* test: *U* = 181.0, *Z* = − 2.32, *P* = 0.021). That was most likely due to larger colony size in less-disturbed forests, as we observed a positive correlation between colony size and overall brood production (sum of number of larvae, pupae, and freshly eclosed callow workers) in both habitat types (Fig 4), for less-disturbed and more-disturbed forests: *rs* = 0.620, *P* = 0.042, and *rs* = 0.588, *P* < 0.0001, respectively).

## Discussion

Our study demonstrates that even species that can adapt to disturbed conditions suffer from diminished colony size and decreased brood production due to human-induced disturbance. Furthermore, it should consider the possibility of hindered factor such as general reduction of habitat productivity producing smaller wood pieces and fewer food sources for ant colonies. In more-disturbed forests, ants formed threefold smaller colonies (Fig 2a) than in less-disturbed locations, most likely due to a lack of suitable nest sites for large colonies (Fig 3) as indicated by our multi-regression model. Large colonies of *P. punctata* in all of the study locations were found in large pieces of hard and dry wood for nesting, which are absent in more-disturbed habitats (Fig 3). Thus, disturbance of rain forest leads to reduction of nest site size, resulting in reduced colony size. Such decreased colony size in disturbed locations correlates with diminished brood production as number of produced brood is proportional to colony size (Fig 4). Smaller colony size can decrease colony survival (Johnson 2004) and, along with lower brood production, may increase the probability of species extinction in human-disturbed habitats due to stochastic events, such as hurricanes. Therefore, reduction of nest site size in disturbed habitats, which leads to diminished colony size and lower brood production, can be a mechanistic explanation why, in consequence, species number decreases in human-disturbed tropical forests. Indeed, in our study locations, the smaller size of wood piece inhabited by an average ant colony may provide less-convenient shelter and poorer protection against rain or larger predators, such as Puerto Rican Woodpecker (*Melanerpes portoricensis*), and therefore lead to lower colony survival in more-disturbed habitats. Moreover, smaller colony size weakens populations in disturbed habitats further due to diminished brood production in small colonies. Indeed, small colonies that were typical for more-disturbed locations produced significantly less brood than colonies from less-disturbed locations (Fig 2b). Alternatively, less large nest sites but many small sites in the more-disturbed area may result in a larger number of colonies and overall a large population size even if the average colony size is smaller. That most likely makes colonies from disturbed habitats even more prone to local extinction as low brood production decreases their ability to stabilize or increase the number of colonies in next generations. Our results concur with previous observations in other social insects that small colonies produce less (Luque *et al* 2013) or even any sexual brood (Walter *et al* 2011). Our field observations are also consistent with results of a laboratory experiment (Hartmann *et al* 2003) where *P. punctata* colonies with experimentally added larger number of larvae, but otherwise with size similar to that found in our disturbed habitats, were not able to rear surplus brood. We documented that a large size of hollowed wood, along with high hardness and low moisture, make a wood piece more favorable for colonization. Thus, habitat disturbance, caused by removal of large trees from a plot by logging or clear-cutting, leads to further reduction of potential nest sites, as hard and dry wood is already limited in rain forest due to high rainfall and high decomposition rate. Effect of both size and other characteristics of wood inhabited by the ants supports the hypothesis that availability of nest sites influences ant colony size in disturbed rain forest. Our results concur with studies indicating the importance of availability of nest sites for animal biodiversity (Tews *et al* 2004) and ants in particular (Schulz & Wagner 2002, Powell *et al* 2011 Klimes *et al* 2012, Yanoviak *et al* 2012, Michlewicz & Tryjanowski 2017).

In conclusion, the results indicate that human-induced disturbance results in reduced average size of available nest sites in tropical rain forests, and that leads to significantly diminished ant colony size and brood production. The smaller population size and lessened reproduction likely makes populations inhabiting disturbed habitats more vulnerable to extinction than populations inhabiting undisturbed habitats. Thus, in addition to the well-documented loss of species richness in human-disturbed tropical habitats (Tews *et al* 2004, Klimes *et al* 2012), we demonstrated the direct effect of the disturbance that may promote vulnerability of local populations. That emphasizes importance of protection of undisturbed tropical rain forests and provides further argument for their comprehensive protection (Myers *et al* 2000, Brooks *et al* 2006, Gibson *et al* 2011).We hope that our study will encourage scientists worldwide to study factors that promote extinction of local animal populations in habitats disturbed by human activity.

## Electronic supplementary material

ESM 1 (DOC 44 kb)
