# Supplementary material for: Ants Response to Human-Induced Disturbance in a Rain Tropical Forest
Source: Neotrop Entomol. 2018 Aug 23;47(6):757–62. doi: 10.1007/s13744-018-0624-5 (PMC6267389; doi:10.1007/s13744-018-0624-5)
Supplement: Supplementary file 1 — (DOC 44 kb) [file 13744_2018_624_MOESM1_ESM.doc]

**Supplementary Material.** Undisturbed and disturbed collecting sites in Puerto Rico (August 2011): type of human-induced disturbance, land use history type of forest; DBH = diameter at breast height.

| Collecting site | Coordinates | Area (ha) | Type of humaninduced disturbance | Type of forest (Helmer *et al.* 2002); vegetation in 2011 |
| --- | --- | --- | --- | --- |
| El Verde 1 | 18°19'17.52"N 65°49'7.08"W | 0.25 | Without humaninduced disturbance since 1934; protected as part of El Yunque National Forest; used as coffee plantation before 1934 | Lower montane wet evergreen forest or “Tabacuno” forest; regrown mature forest with understory and epiphytes |
| El Verde 2 | 18°19'13.73"N 65°49'7.53"W | 0.25 | Without humaninduced disturbance since 1934; protected as part of El Yunque National Forest; used as coffee plantation before 1934 | Lower montane wet evergreen forest or “Tabacuno” forest; regrown mature forest with understory and epiphytes |
| El Verde 3 | 18°19'12.31"N 65°49'5.71"W | 0.25 | Without humaninduced disturbance since 1934; protected as part of El Yunque National Forest; used as coffee plantation | Lower montane wet evergreen forest or “Tabacuno” forest; regrown mature forest with understory and epiphytes |
| El Verde 4 | 18°19'10.63"N 65°49'3.74"W | 0.25 | Without humaninduced disturbance since 1934; protected as part of El Yunque National Forest; used as coffee plantation before 1934 | Lower montane wet evergreen forest or “Tabacuno” forest; regrown mature forest with understory and epiphytes |
| El Verde 5 | 18°19'11.75"N 65°49'2.06"W | 0.25 | Without humaninduced disturbance since 1934; protected as part of El Yunque National Forest; used as coffee plantation before 1934 | Lower montane wet evergreen forest or “Tabacuno” forest; regrown mature forest with understory and epiphytes |
| El Verde 6 | 18°19'13.05"N 65°48'59.82"W | 0.25 | Without humaninduced disturbance since 1934; protected as part of El Yunque National Forest; used as coffee plantation before 1934 | Lower montane wet evergreen forest or “Tabacuno” forest; regrown mature forest with understory and epiphytes |
| El Verde 7 | 18°19'14.93"N 65°49'3.43" W | 0.25 | Without humaninduced disturbance since 1934; protected as part of El Yunque National Forest; used as coffee plantation before 1934 | Lower montane wet evergreen forest or “Tabacuno” forest; regrown mature forest with understory and epiphytes |
| El Verde 8 | 18°19'53.77"N 65°49'20.23"W | 0.25 | Without humaninduced disturbance since 1934; protected as part of El Yunque National Forest; used as coffee plantation before 1934 | Lower montane wet evergreen forest or “Tabacuno” forest; regrown mature forest with understory and epiphytes |
| Juncos | 18°16'35.96"N 65°54'15.80"W | 0.28 | Disturbed; logged in 2004 with central part being clear-cut | Submontane wet evergreen forest; trees and shrubs along road and abandoned field |
| Sabana | 18°19'22.93"N 65°43'8.63"W | 0.27 | Disturbed; logged before 2004 | Submontane wet evergreen forest; trees and shrubs along road and abandoned field |
| Yuquiyu 1 | 18°20'50.92"N 65°43'47.17"W | 0.39 | Disturbed; used as a pasture, trees thicker than 0.1 m are cut on BH and removed ever few years, clear-cut in 2003 | Lowland moist seasonal evergreen forest/shrub; open forest with young trees (> 0.1 m DBH) growing 1-5 m from each other, plot with one mature tree (> 0.3 m DBH) |
| Yuquiyu 2 | 18°20'37.38"N 65°43'40.60"W | 0.37 | Disturbed; extensive logging, property used as household before 1981 | Lowland moist seasonal evergreen forest/shrub; dense young forest |
| Liquillo 1 | 18°22'51.74"N 65°43'51.56"W | 0.49 | Disturbed; extensive logging, formerly used as a pasture (mid 1980’s) | Moist seasonal evergreen forest; open young forest with medium size trees (0.2-0.3 m DBH) and dense understory (trees > 0.07 m DBH) |
| Liquillo 2 | 18°22'54.34"N 65°43'40.66"W | 0.41 | Disturbed; extensive logging, used as pasture with trees before 2004 | Lowland moist seasonal evergreen forest and lowland moist coconut palm forest; coconut palms with understory formed by thin (>0.05 m DBH) young trees |
| Parcelas Vieques | 18°25'3.99"N 65°49'40.93"W | 1.76 | Disturbed; extensive logging | Lowland moist coconut palm forest and seasonally flooded evergreen forest and; coconut palms with addition of medium size trees (0.2-0.3 m DBH) and dense understory (trees > 0.07 m DBH) |
| Pitahaya | 18°21'6.45"N 65°42'13.86"W | 0.075 | Disturbed; regrow forest patch on plot used as a pasture before 1995 | Lowland moist seasonal evergreen forest/shrub; forest patch (20 m wide) between road and pasture, dense forest with young trees (> 0.1 DBH) |
| El Tunel | 18°28'57.51"N 66°57'54.69"W | 0.24 | Disturbed; used as a pasture before 1993; extensive logging after 1993 | Lowland dry semidecidous forest; regrown young forest (trees with > 0.1 DBH) |
| Pico | 18° 6'19.27"N 67° 2'13.19"W | 1.57 | Disturbed; used as pasture before 2005 | Lowland moist seasonal evergreen forest/shrub; dense forest with young trees (> 0.1 DBH) |
